# Crevicular Alkaline Phosphatase Activity and Rate of Tooth Movement of Female Orthodontic Subjects under Different Continuous Force Applications

**DOI:** 10.1155/2013/245818

**Published:** 2013-05-02

**Authors:** Rohaya Megat Abdul Wahab, Maryati Md Dasor, Sahidan Senafi, Asma Alhusna Abang Abdullah, Zulham Yamamoto, Abdul Aziz Jemain, Shahrul Hisham Zainal Ariffin

**Affiliations:** ^1^Department of Orthodontics, Faculty of Dentistry, Universiti Kebangsaan Malaysia, 50300 Kuala Lumpur, Malaysia; ^2^Department of Orthodontics, Faculty of Dentistry, Universiti Teknologi MARA, 40450 Shah Alam, Selangor, Malaysia; ^3^School of Bioscience and Biotechnology, Faculty of Science and Technology, Universiti Kebangsaan Malaysia, 43600 Bangi, Selangor, Malaysia; ^4^School of Mathematics, Faculty of Science and Technology, Universiti Kebangsaan Malaysia, 43600 Bangi, Selangor, Malaysia

## Abstract

*Purpose*. This study is aimed to compare the effects of two different orthodontic forces on crevicular alkaline phosphatase activity, rate of tooth movement, and root resorption. *Materials and Methods*. Twelve female subjects of class II division 1 malocclusion participated. Maxillary canines with bonded fixed appliances acted as the tested teeth, while their antagonists with no appliances acted as the controls. Canine retraction was performed using nickel titanium coil spring that delivered forces of 100 gm or 150 gm to either side. Crevicular fluid was analyzed for ALP activity, and study models were casted to measure tooth movements. Root resorption was assessed using periapical radiographs before and after the force application. *Results*. ALP activity at the mesial sites peaked at week 1 for 150 gm group with significant differences when compared with the 100 gm group. Cumulative canine movements were significantly greater in the 150 gm force (2.10 ± 0.50 mm) than in the 100 gm force (1.57 ± 0.44 mm). No root resorption was in the maxillary canines after retraction. *Conclusions*. A force of 150 gm produced faster tooth movements and higher ALP activity compared with the 100 gm group and had no detrimental effects such as root resorption.

## 1. Introduction

Orthodontia is based on the application of prolonged forces on teeth. Various degrees of force magnitude, frequency, and duration of orthodontic treatment exert a great influence on the surrounding tissue reaction and bone modeling [1]. Alveolar bone modeling during orthodontic tooth movement is a continuously balanced process between bone formation and bone resorption [2]. In the concepts of bone physiology, bone modeling involves the change of shape in the bone, while bone remodeling is a couple process of resorption and deposition resulting in bone turnover but not a gross change in the bone morphology [3]. 

Interaction between bone formation and resorption during tooth movement results in the release of various biochemical or cellular mediators that can be identified as potential biomarkers [1]. Many studies have investigated possible biomarkers for bone modeling during orthodontic tooth movement [4–10]. Bone biomarker such as alkaline phosphatase enzyme (ALP) has often been associated with bone formation [6, 11–13]. Higher ALP activity has been detected at tension sites compared with compression sites during orthodontic tooth movement [4]. 

Gingival crevicular fluid (GCF) is an osmotically mediated inflammatory exudate found in the gingival sulcus. Obtaining GCF samples is a noninvasive, relatively simple, and easily repetitive procedure with minimal risk imposed on the patient. Therefore, it was chosen as a mean to obtain samples from orthodontically moved teeth. Changes in the composition of GCF were highly correlated to any changes occurring deep in the periodontium [14]. Hence, the analysis of GCF samples provides a better understanding of the dynamic and metabolic status associated with orthodontic tooth movement [15].

An optimal force is one at certain magnitudes and temporal characteristics that are capable of producing maximum rates of tooth movement and with maximum patient comfort [16]. A systematic review by Ren et al. [16] indicated that there are no consistencies or agreements regarding optimal force levels in clinical orthodontics. Forces involved in canine distalization can range from 100 gm [8] or 150 gm [17, 18] to 200 gm [19, 20]. Samuels et al. [5] found that by increasing the force from 150 gm to 200 gm, similar rates of space closure are produced. However, for this study, forces of 100 gm and 150 gm were compared in terms of ALP activity and the rate of tooth movement. Furthermore, the possible detrimental effects of different orthodontic forces such as root resorption were taken into consideration.

The objective of this study was to compare the effects of different orthodontic forces (100 gm or 150 gm) on specific ALP activities in GCF and their relationship to the rate of canine movement during five weeks of retraction. This study also aimed to observe root resorption for six months after retraction.

## 2. Materials and Methods

### 2.1. Patients Selection

All patients were selected based on the inclusion criteria as stated in Table 1. All patients were prohibited from taking any anti-inflammatory drugs throughout the study period as they may interfere with the tooth movement [21]. Prophylaxis treatments were done to all patients to ensure optimal oral health four weeks prior to the study. Informed consents were obtained from all the participants or guardians (for patients under 16 years of age). Ethical approvals were obtained from the Research Ethical Committee of *Universiti Kebangsaan Malaysia* (no. 1.5.3.5/244/DD/034 (1)/2009).

### 2.2. Orthodontic Appliances and Experimental Teeth

A Nance appliance was fitted to the maxillary first molars prior to the extractions. The buccal surfaces of the maxillary teeth (e.g., incisors, canines, and premolars) were bonded with a 0.022 × 0.028-inch preadjusted edgewise appliance (American Orthodontics, Mini Master; MBT prescription). The alignment stage was started with a 0.014-inch NiTi archwire and completed with 0.018 × 0.025-inch NiTi archwire within three to four consecutive reviews. The working archwire of 0.019 × 0.025-inch SS was inserted and left *in situ* for four weeks to allow passivity between the archwire and the bracket's slot before initiating canine retraction. Both of the maxillary canines acted as the test teeth, while their antagonists with no orthodontic appliance acted as the control teeth. 

Canine retraction was performed by placing a light NiTi push coil spring (sds Ormco) between maxillary lateral incisors and canines. A 0.019-inch SS ligature was used to ligate all incisors together and to achieve individual ligation at lateral incisors and canines. In a split-mouth design, patients received a 100 gm or 150 gm force either on the right or left side of the maxillary arch via the “toss of a coin” technique. The forces were measured using a Correx gauge (dial-type stress and tension gauge; Dentaurum, Germany). Patients were reviewed, and GCF was collected on a weekly basis for six consecutive weeks. GCF that was collected before the application of force served as the baseline.

### 2.3. GCF Collection and Alkaline Phosphatase Assay

The maxillary canines were isolated using cotton rolls and were gently dried for 5 s. Consequently, the native GCF was extracted using methylcellulose filter paper strips (Periopaper, Proflow, Amityville, NY) at the mesial and distal sides of the test and control teeth. Each strip was inserted 1 mm in the gingival crevice and left *in situ* for 60 s while maintaining isolation. A total of three strips were used at intervals of 60 s to maximize the volume of GCF collected per site [22]. All strips were inserted into 1.5 mL Eppendorf tubes containing 80 *μ*L of physiologic saline and were centrifuged for 5 minutes at 4000 ×g using a microcentrifuge machine (Hettich Zentrifugen Mikro 22R) to completely elute the GCF components. The supernatant was immediately analyzed.

Enzyme activity was determined using a spectrophotometer at 405 nm (Varian Cary 50UV-Vis). The GCF samples of 50 *μ*L were incubated for 30 minutes at 30°C in a substrate containing 50 *μ*L of *ρ*-nitrophenyl phosphate (10 mmol/L), 250 *μ*L of carbonate buffer (pH 9.8), 50 *μ*L of mannitol (200 mmol/L), 50 *μ*L of MgCl_2_ (3 mmol/L), and 0.1 mL of sterile distilled water. Water was added to increase the total volume to 0.5 mL. Enzyme activity was then terminated by the addition of 0.7 mL NaOH (4 M) to the component (sample and substrate). Immediately, the absorbance (in optical density) was measured in a spectrophotometer. Standard curve used is 1 mM of *ρ*-nitrophenol solution. The absorbance is converted into enzymatic activity unit (1 U = 1 *μ*mol of *ρ*-nitrophenol released per minute at 30°C). The ALP-specific activities were determined based on units (Us) of activity per total protein content [23] and were stated as U/mg. A standard curve of bovine serum albumin (Sigma, USA) protein was prepared earlier to determine the total protein content [23] for each assay.

### 2.4. Canine Movement and Evaluation of Periapical Radiographs

Study models were fabricated at every visit to measure canine movement. Canine movement was measured from the distal margin of the lateral incisor bracket to the mesial margin of the canine bracket. Measurements were made using a digital caliper (KERN, Germany) with a sensitivity of ±0.01 mm. Cumulative canine distances were obtained at the end of the experimental term. The periapical radiographs for the test and control teeth were taken preoperatively, prior to the placement of the NiTi coil springs and also six months after retraction. These periapical radiographs were projected on a screen and magnified tenfold. They were assessed for apical and lateral surface root resorptions by the following scores listed in Table 2, adopted from the methods reported by Liou and Huang [24]. However, we have the false sense of accuracy when using an algorithm method of measuring root resorption on serial radiographs [25].

### 2.5. Statistical Analysis

The data was analyzed statistically using SPSS version 20. Normality distribution of the ALP activities data was measured using the Kolmogorov-Smirnov test. The independent Student's *t*-test was used to compare the ALP activities between the test teeth group and control teeth group. The paired *t*-test was used to compare ALP activity to the respective baseline value weekly. The comparisons of cumulative canine movements (mm) with times (week) were analyzed using the paired *t*-test and correlation test.

## 3. Results

A total of twelve healthy female orthodontic patients with ages ranging from 14 to 28 years completed this study. The mean age of the participants was 24.7 ± 3.0 years. In the 150 gm group, ALP activity at baseline showed no significant differences between the test and control sites (*P* > 0.05) (Table 3). Peak ALP activity was noted at the mesial sites of the test canines at week 1 under 150 gm force, which was three times significantly higher (*P* < 0.05) than ALP activity in control teeth (Table 3(A)). ALP activities of test canines at weeks 1 and 2 were also significantly higher when compared with ALP activity at baseline (Table 3(A)). ALP activity was stabilised throughout the following weeks. On the other hand, ALP activity decreased over four consecutive weeks at the distal sites of the test teeth, and no statistical significant differences (*P* > 0.05) were observed (Table 3(A)). 

The pretreatment baseline ALP activities with the 100 gm force also were not significantly different (*P* > 0.05) from the control teeth at both sites. At week 2, ALP activity was at its peak compared with the baseline at the mesial sites (Table 3(B)). The peak enzyme activity of the test site was 2.5 times higher than baseline activity, though it showed no statistically significant differences (*P* > 0.05) (Table 3(B)). ALP activity later showed a fall at week 3 and stabilised 2 weeks later. Furthermore, there were no significant differences (*P* > 0.05) noted in enzyme activity between the test and control teeth at the distal sites (Table 3(B)).

The specific ALP activities were also compared between the two orthodontic forces (100 gm versus 150 gm). ALP activity was significantly higher with the 150 gm force at week 1 (*P* < 0.05) at the mesial sites than with the 100 gm force (Table 3(C)). At the distal sites, there were no significant differences (*P* > 0.05) between the two orthodontic forces (Table 3(C)). At the mesial sites, the specific ALP activity with the 150 gm force later decreased by 33% at week 2, while it decreased by 60% at week 3 with the 100 gm force group (Table 3(A) and 3(B)). The remaining weeks showed no significant difference (*P* > 0.05) in enzyme activity between the 150 gm and 100 gm force groups at the mesial sites. 

**Figure 1:**
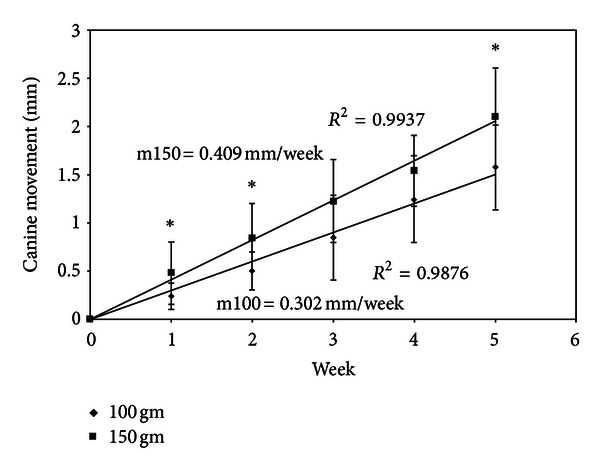
Comparison between movements of distalized maxillary canines with 100 gm and 150 gm orthodontic forces over five consecutive weeks. *Significant (*P* < 0.05).

There was a linear relationship between cumulative canine movement (mm) with time (week) in both the 100 gm and 150 gm force groups (Figure 1). There was a significantly faster rate of canine movement in the 150 gm force group in the first week of canine movement (*P* < 0.05) than with 100 gm. However, no significant (*P* > 0.05) canine movement was observed in the following weeks when the two force groups were compared (Table 4(A)). The maxillary canines with 150 gm of force moved 25% significantly faster than those with 100 gm force (*P* < 0.05) (Figure 1), where the mean cumulative canine movement was 2.10 ± 0.50 mm with 150 gm of force with a rate of 0.409 mm/week and 1.57 ± 0.44 mm with 100 gm of force with a rate of 0.302 mm/week (Table 4(B)). 

**Figure 2:**
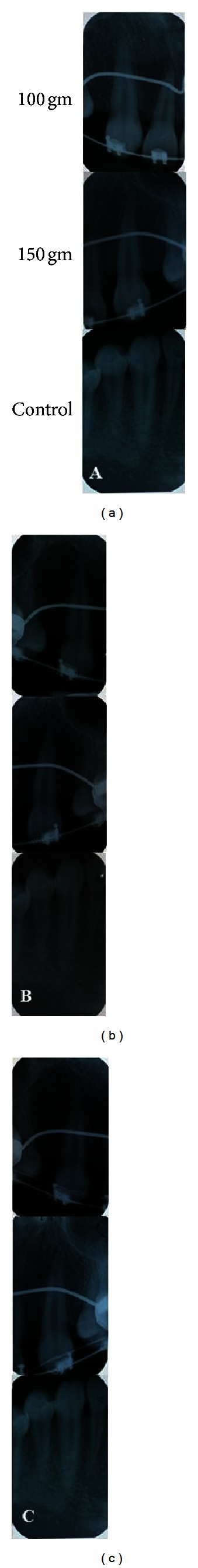
Periapical radiographs of canine test teeth for 100 gm and 150 gm groups and control teeth. Apical and lateral root resorptions were assessed using periapical radiographs taken at baseline (a), five weeks (b), and six months after canine retraction (c).

Signs of canine root resorption, monitored using periapical radiographs, showed that there were no lateral or apical root resorptions (score 0) in the canines in either the 150 gm or 100 gm orthodontic force groups, as well as in the control groups (Figure 2). Overall, the mesial sites associated with 150 gm force showed significantly higher ALP activity than did the distal sites (*P* < 0.05) (Table 3(A)). A NiTi coil spring exerting a 150 gm force produced significantly faster canine movement than did the 100 gm force (*P* < 0.05) (Figure 1). Moreover, there were no root resorptions detected around the canines for either the test or the control teeth between the two different orthodontic forces (Figure 2).

## 4. Discussion

This prospective study was designed to investigate the relationship between orthodontic forces and rate of tooth movement at a weekly basis based on ALP activity in GCF. The results of this study showed statistically significant (*P* < 0.05) increases in ALP activity at the mesial sites of test teeth at week 1 (Table 3(A)) and faster cumulative canine movement in week 5 with 150 gm of force (Figure 1). These findings are in females and may not be the same in males. They may or even likely apply to males as well, but we do not know that, and we present no data to support that presumption.

Many past clinical studies have sought to search for the most optimal force for orthodontic tooth movement. Batra et al. [26] have suggested 100 gm to be the optimal force. However, another study has suggested 150 gm to 200 gm as a better force in moving teeth without anchorage loss [27]. Samuels et al. [5] did a comparative study on all three forces: 100 gm, 150 gm, and 200 gm. Among the three forces, 150 gm was suggested to be the most effective force for tooth movement. In the present study, 150 gm force produced faster tooth movement than 100 gm force. 

In the bone modeling process, bone formation occurs between the first and second weeks at sites of both tension and pressure. Bone formation has been shown to be represented by the expression of ALP [10]. In our study, ALP peaked at week 1 in the 150 gm group and at week 2 in the 100 gm group. Similar time ranges were observed by Insoft et al. [28], where the researchers observed that ALP peaked between weeks 1 and 3. Batra et al. [26] reported similar findings, where they observed that ALP peaked at week 2 using 100 gm of force.

The reductions in the ALP activities were seen at weeks 2 (150 gm) and 3 (100 gm). The decrease in ALP activity was related to the hyalinized zone, which is subsequently removed by osteoclasts [29]. Similar patterns were also observed in a study by Asma et al. [18]. Enzyme activity was found to be negatively correlated to the rate of tooth movement according to specific times and sites [18]. At the pressure sites of the test teeth, bone resorption was more commonly observed than bone formation. However, in our five weeks of observation, overall ALP activity increased with greater rates of tooth movement with 150 gm of force. This phenomenon in our study may reflect the contribution of osteoblastic activities during both bone formation and resorption.

Anchorage reinforcements were done using Nance appliances in posterior directions and while the ligation of incisors was done in anterior directions. These procedures were implemented as per Yee et al. [27], who reported posterior anchorage losses during canine retraction under heavy forces of 300 gm. Canine retraction was done on a rectangular wire (0.019 × 0.025-inch SS) to promote more bodily movement than tipping movement that is observed with a round wire (0.016-inch AJ Wilcock SS). 

In this study, we used a spectrophotometer to analyse ALP activity. GCF samples were obtained three times, and optimisations of the samples were performed to ensure true representation of ALP activity. Comparisons of ALP activities were done between the test and the antagonist control teeth because the latter represented normal physiological bone remodeling under normal masticatory forces. 

The duration of 5 weeks after force application and weekly intervals for sample collection was formulated to monitor ALP patterns and to understand the enzymatic changes occurring as a result of alveolar bone changes during bone modeling. Some studies have looked at ALP activity on a monthly basis [10]. It was observed that ALP activity started to stabilise towards the latter parts of the month, which means that ALP activity will not be detected if it is measured on a monthly basis. In orthodontics, patients are routinely reviewed for orthodontic appliance activation between four and six weeks. This study found that ALP activity was at its peak at weeks 1 and 2 for 150 gm and 100 gm of force, respectively. On the basis of these results, bone formation occurred earlier and more rapidly in orthodontic tooth movements associated with higher forces (150 gm), which help to stabilise teeth in new orthodontic position. 

Root resorption is one of the detrimental effects resulting in orthodontic treatment. For that reason, periapical radiographs were taken before and after the application of force to identify any signs of apical and lateral root resorptions around the maxillary canines after retraction. Periapical radiographs provide more accurate views of the alveolar bone and root compared with panoramic radiographs in assessing root resorption and vertical bone loss. The later radiograph overestimates the amount of root resorption by 20% or more [30]. This study is adopted from Liou and Huang's methodology for the assessment of root resorption [24]. In addition, a study showed that any root resorption can be identified within six months [31]. Therefore, our study monitored root resorption for six months and found neither apical nor lateral root resorptions of the retracted canines.

## 5. Conclusions

Orthodontic forces of 150 gm produced 25% faster tooth movements, as indicated by the significant increases in ALP activity at week 1 during the canine retraction stage compared with the 100 gm group (*P* < 0.05). A force of 150 gm had no detrimental effects such as root resorption detected six months after retraction.

## Figures and Tables

**Table 1:** The inclusion criteria for patient selection.

Inclusion criteria	
(1) Healthy with no known systemic diseases	
(2) Good general and periodontal health and not pregnant	
(3) Mild-to-moderate crowding of the maxillary and mandibular arches	
(4) Need at least maxillary first premolar extractions	
(5) Canine relationship of class II 1/2 unit or more	
(6) Class II/1 incisal relationship with an overjet of more than 6 mm	
(7) Overbite not more than 50%	
(8) No use of any anti-inflammatory drugs during the study	
(9) No previous orthodontic or orthopaedic treatment	
(10) No craniofacial anomalies	

**Table 2:** The assessment scores for apical and lateral root resorption using periapical radiographs (17).

Score	Apical root resorption	Lateral root resorption
0	No apical root resorption	Smooth lateral root surface and periodontal ligament

1	Slight blunting of the canine root apex	Slightly irregular lateral root surface; not beyond one-third of the dentine width between the distal-side periodontal ligament and pulp chamber

2	Moderate resorption of the root apex beyond blunting and up to one-fourth of the root length	Moderate irregular lateral root surface beyond one-third and up to two-thirds of the dentine width between the distal-side periodontal ligament and pulp chamber

3	Excessive resorption of the root apex beyond one-fourth of the root length	Excessive irregularity of the lateral root surface beyond two-thirds of the dentine width between the distal-side periodontal ligament and pulp chamber

**Table 3:** GCF ALP activities on the tension (mesial) and compression (distal) sites of distalized maxillary canines as test tooth (TT) and mandibular canines as control tooth (CT) under 100 gm and 150 gm of orthodontic forces. Data were presented as mean and standard deviation (SD).

		(A) Force 150 gm					(B) Force 100 gm					(C) Force 100 gm versus 150 gm
	Time	TT	CT	Independent *t*-test	Paired *t*-test		TT	CT	Independent *t*-test	Paired *t*-test		Paired *t*-test
					TT	CT				TT	CT	TT
	Baseline	2.76 SD 1.83	2.21 SD 1.13	NS	—	—	3.02 SD 2.14	4.67 SD 8.39	NS	—	—	NS
	Week 1	9.71 SD 6.31	3.31 SD 2.16	*Sig *	*Sig *	NS	5.30 SD 4.09	5.94 SD 6.06	NS	NS	NS	*Sig *
Mesial	Week 2	6.43 SD 4.99	4.26 SD 3.99	NS	*Sig *	NS	8.07 SD 10.72	3.17 SD 2.13	NS	NS	NS	NS
	Week 3	4.69 SD 3.32	3.38 SD 2.10	NS	NS	NS	3.15 SD 1.32	2.48 SD 1.50	NS	NS	NS	NS
	Week 4	4.55 SD 2.72	3.18 SD 2.69	NS	NS	NS	3.20 SD 1.38	4.69 SD 5.22	NS	NS	NS	NS
	Week 5	4.74 SD 3.29	3.51 SD 2.04	NS	NS	NS	4.28 SD 4.72	2.78 SD 1.50	NS	NS	NS	NS

	Baseline	4.77 SD 4.43	2.91 SD 1.68	NS	—	—	3.44 SD 2.46	3.10 SD 2.65	NS	—	—	NS
	Week 1	4.47 SD 2.60	5.40 SD 3.85	NS	NS	NS	4.70 SD 3.06	7.98 SD 9.54	NS	NS	NS	NS
Distal	Week 2	3.15 SD 2.85	2.32 SD 1.02	NS	NS	NS	3.37 SD 2.70	3.22 SD 1.42	NS	NS	NS	NS
	Week 3	3.36 SD 2.83	3.72 SD 2.32	NS	NS	NS	3.83 SD 2.88	4.04 SD 3.06	NS	NS	NS	NS
	Week 4	3.31 SD 1.77	3.35 SD 2.04	NS	NS	NS	4.00 SD 3.37	3.30 SD 2.29	NS	NS	NS	NS
	Week 5	4.26 SD 2.84	3.31 SD 1.74	NS	NS	NS	4.99 SD 3.44	3.79 SD 2.03	NS	NS	NS	NS

NS: no statistically significant difference. *Sig*: statistically significant difference.

**Table 4:** Comparisons between the measurement of canine movements at 150 gm and 100 gm orthodontic forces using a paired *t*-test.

Time	Continuous orthodontic force		*P* value
	150 gm	100 gm	
(A) Canine movement			
Week 1 (W1-W0)	0.48 ± 0.32	0.24 ± 0.14	0.01*
Week 2 (W2-W1)	0.36 ± 0.30	0.25 ± 0.15	0.17
Week 3 (W3-W2)	0.38 ± 0.31	0.36 ± 0.30	0.86
Week 4 (W4-W3)	0.33 ± 0.21	0.39 ± 0.24	0.41
Week 5 (W5-W4)	0.55 ± 0.55	0.33 ± 0.17	0.20
(B) Cumulative canine movement			
Week 1	0.48 ± 0.32	0.24 ± 0.14	0.01*
Week 2	0.84 ± 0.36	0.50 ± 0.20	0.01*
Week 3	1.22 ± 0.43	0.85 ± 0.44	0.06
Week 4	1.54 ± 0.37	1.24 ± 0.45	0.13
Week 5	2.10 ± 0.50	1.57 ± 0.44	0.04*

Significance = *P < 0.05.

Data presented as mean ± standard deviation of canine movement in a week (*n* = 12) with unit of mm.
